# Magnetic resonance imaging of the time course of hyperpolarized ^129^Xe gas exchange in the human lungs and heart

**DOI:** 10.1007/s00330-018-5853-9

**Published:** 2018-12-05

**Authors:** Ozkan Doganay, Mitchell Chen, Tahreema Matin, Marzia Rigolli, Julie-Ann Phillips, Anthony McIntyre, Fergus V. Gleeson

**Affiliations:** 1 Department of Oncology, University of Oxford, Old Road Campus Research Building, Roosevelt Drive, Oxford, OX3 7DQ UK; 2 Department of Radiology, The Churchill Hospital, Oxford University Hospitals NHS Trust, Old Rd, Oxford, OX3 7LE UK; 3 University of Oxford Centre for Clinical Magnetic Resonance Research, Division of Cardiovascular Medicine, Radcliffe Department of Medicine, John Radcliffe Hospital, Headley Way, Oxford, OX3 9DU UK

**Keywords:** Magnetic resonance imaging, Xenon, Pulmonary emphysema, Chemical shift imaging, Heart

## Abstract

**Purpose:**

To perform magnetic resonance imaging (MRI), human lung imaging, and quantification of the gas-transfer dynamics of hyperpolarized xenon-129 (HPX) from the alveoli into the blood plasma.

**Materials and methods:**

HPX MRI with iterative decomposition of water and fat with echo asymmetry and least-square estimation (IDEAL) approach were used with multi-interleaved spiral k-space sampling to obtain HPX gas and dissolved phase images. IDEAL time-series images were then obtained from ten subjects including six normal subjects and four patients with pulmonary emphysema to test the feasibility of the proposed technique for capturing xenon-129 gas-transfer dynamics (XGTD). The dynamics of xenon gas diffusion over the entire lung was also investigated by measuring the signal intensity variations between three regions of interest, including the left and right lungs and the heart using Welch’s *t* test.

**Results:**

The technique enabled the acquisition of HPX gas and dissolved phase compartment images in a single breath-hold interval of 8 s. The *y*-intersect of the XGTD curves were also found to be statistically lower in the patients with lung emphysema than in the healthy group (*p* < 0.05).

**Conclusion:**

This time-series IDEAL technique enables the visualization and quantification of inhaled xenon from the alveoli to the left ventricle with a clinical gradient strength magnet during a single breath-hold, in healthy and diseased lungs.

**Key Points:**

• *The proposed hyperpolarized xenon-129 gas and dissolved magnetic resonance imaging technique can provide regional and temporal measurements of xenon-129 gas-transfer dynamics.*

*• Quantitative measurement of xenon-129 gas-transfer dynamics from the alveolar to the heart was demonstrated in normal subjects and pulmonary emphysema.*

*• Comparison of gas-transfer dynamics in normal subjects and pulmonary emphysema showed that the proposed technique appears sensitive to changes affecting the alveoli, pulmonary interstitium, and capillaries.*

**Electronic supplementary material:**

The online version of this article (10.1007/s00330-018-5853-9) contains supplementary material, which is available to authorized users.

## Introduction

Hyperpolarized xenon-129 (HPX) magnetic resonance imaging (MRI) may be used to evaluate pulmonary ventilation, in patients with respiratory diseases such as chronic obstructive pulmonary disease (COPD) and asthma [1–8]. Due to the solubility of xenon, HPX MRI may also provide additional functional information about the gas exchange efficiency in the alveolar epithelium, the interstitium, the pulmonary capillary endothelium, and the blood plasma of the lungs [9]. At clinical MR magnetic field strengths (1.5–3 T), the dissolved xenon gas splits into two chemical shifts corresponding to the pulmonary tissue and blood plasma (PTBP) and red blood cell (RBC) compartments of the lung. The vast majority of the previous HP dissolved xenon studies focused on the measurement of diffusion uptake (i.e., gas-transfer dynamics) from xenon in the alveoli into the PTBP and RBC compartments of the entire thoracic cavity using MR spectroscopy techniques [10–12]. These techniques allow measurement of lung function when used in corroboration with numerical modeling [13–17]. Recent HPX MRI techniques have enabled the spatial distribution of regional diseases, addressing the challenges arising from low signal-to-noise ratios (SNR) and short transverse signal decay time (*T*_2_^*^ ≈ 2 ms) of PTBP and RBC peaks [18–23]. Concurrent imaging of the dissolved phases of xenon and the measurement of gas-transfer dynamics would enable quantitative information about the efficiency or otherwise of gas exchange pulmonary function.

An early dissolved phase imaging technique, the xenon polarization transfer constant (XTC) has been shown to be sensitive to measurement of gas-transfer dynamics combining with the spatial information [24, 25]. In contrast to MR spectroscopy, XTC enabled the indirect measurement of the dissolved phase signal from the estimation of depolarization of the reservoir gas-phase magnetization with respect to various delay times [26, 27]. Unlike other dissolved phase imaging techniques, XTC did not provide imaging of PTBP and RBC compartments of the lungs separately.

MR imaging with iterative decomposition of water and fat with echo asymmetry and least-square estimation (IDEAL) Cartesian k-space sampling has been reported to be an accurate technique for imaging the chemical shift of hydrogen protons in muscle and fat compartments [28]. The IDEAL technique has been used in the metabolic imaging of hyperpolarized (HP) ^13^C using a single-shot spiral k-space sampling, taking advantage of the relatively longer *T*_2_^*^ (*T*_2_^*^ ≈ 30 ms) [29]. This IDEAL with spiral k-space sampling technique was subsequently applied to HPX MR for imaging gas, PTBP, and RBC compartments in rat lungs [30]. The short *T*_2_^*^ of ^129^Xe in the PTBP and RBC compartments was compensated by using an insertable high-performance gradient system (maximum gradient amplitude, 300 mT/m; slew rate, 2500 T/m/s) [31, 32]. While this strategy provided quantitative measurement of the physiological parameters and dynamics of HPX gas diffusion from alveoli air sacs to the heart in a rat model [31, 32], it was difficult to obtain sub-centimeter pixel resolution in humans using this single-shot spiral sampling technique due to the limited gradient performance (maximum gradient amplitude, 50 mT/m; slew rate, 200 T/m/s) of clinical MR scanners.

In this study, HPX MRI single-shot k-space sampling IDEAL technique has now been extended to human lung imaging using multi-interleave spiral k-space sampling in a standard clinical gradient MR scanner. By taking advantage of rapid k-space sampling, the feasibility of capturing the time-series IDEAL gas, PTBP, and RBC compartment images in a single breath-hold across eight different time points has been investigated in six healthy volunteers and four patients with pulmonary emphysema.

## Materials and methods

Six healthy participants (aged 25–45 years) with no known cardiopulmonary disease, no evidence of emphysema or other pulmonary disease on CT, and with normal lung function were scanned using a 1.5 T MR scanner (Signa HDx Twin Speed, GE Healthcare) and a proton-blocked vest-shaped RF coil (Clinical MR solutions). Enriched xenon gas (87% ^129^Xe) was polarized by a commercially available polarizer (Model 9300, Polarean with 10–15% polarization). Subjects were instructed to inhale 1.0 L of pure xenon gas following a protocol approved by the National Research Ethics Service Committee.

**Fig. 1:**
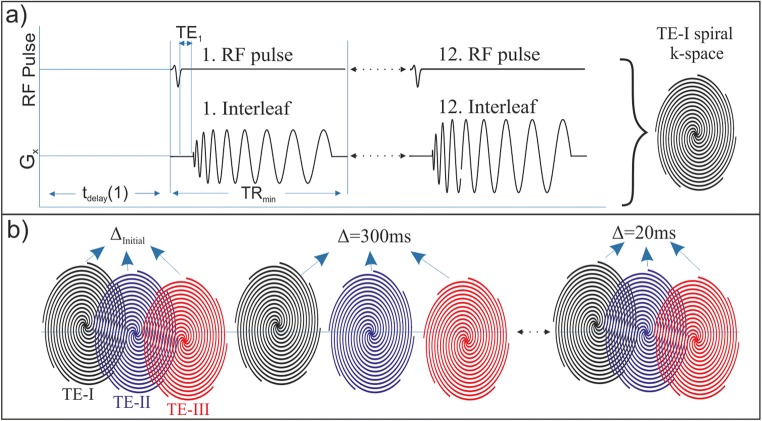
IDEAL pulse sequence timing diagram for the first 12-interleaved echo image (**a**) and time-series IDEAL image (**b**) acquisition diagram with different gas-transfer times, ∆. G_x_ is the readout gradient (G_y_ is identical to G_x_ with 90° off phase and was not shown in a). *t*_delay_ is the additional time delay given for replenishment of xenon dissolved phase signal before each echo image. TR_min_ is the RF pulse repetition time including the RF pulse duration, echo time (TE_1_), k-space readout time per interleaf, and spoiling gradients. TE-I is the echo image, and ∆_initial_ is the spoiling acquisition for destroying initial buildup dissolved phase signal which is accumulated during the time between inhalation of HPX gas bag and the time-series IDEAL images starting

Conventional proton MR 3-plane localizer images were acquired using the standard fast gradient echo sequence and double inversion-recovery (DIR) black-blood imaging sequence to acquire a diagnostic display of the lung, cardiac, and mediastinal anatomy as previously explained [6]. Following the conventional proton scan, IDEAL HPX gas, PTBP, and RBC compartment images were obtained using a multi-interleave spiral k-space with a three-point IDEAL approach. Briefly, three-point IDEAL approach acquisition produced three different echo images (TE-I, TE-II, and TE-III) with three echo times (TE_1_ = 0 μs, TE_2_ = 160 μs, and TE_3_ = 320 μs) which were calculated using the number of signal averaging approach [30]. Each of the echo images were acquired using 12 RF pulses and spiral interleaves for a particular echo time as shown in Fig. 1a using a standard clinical MR gradient. Imaging parameters were field of view. 34 cm; number of pixels, 32; bandwidth, 62.5 kHz; and readout time duration per interleaf, 4 ms. The scan time required for a single echo image was 12 × TR_min_ = 240 ms by setting TR_min_ to the minimum value of 20 ms as shown in Fig. 1a. The TR_min_ consisted of the time required for the slice selection, RF pulse (a hard pulse), the echo times, readout time (~ 4 ms), rewinders, and spoiler gradients per interleaves.

To obtain time-series IDEAL images, the imaging pulse sequence in Fig. 1a was repeated with additional delay times as shown in Fig. 1b. This allowed the acquisition of time-series IDEAL images with various gas-transfer time points, ∆. To investigate the optimum selection of ∆, time-series IDEAL images were acquired from a healthy subject including late ∆ points *∆*_late_: *∆*_initial_, 20, 25, 50, 100, 500, 1000, and 2000 ms leading to a long scan time of ~ 17 s. ∆_initial_ was the spoiling acquisition used to destroy initial buildup of dissolved phase signal which accumulated during the time between the inhalation of HP ^129^Xe gas and the scan starting. To investigate the feasibility of obtaining XGTD, the time-series IDEAL images of four healthy subjects were collected with the following gas-transfer time points: *∆*_short_: *∆*_initial_, 300, 200, 100, 50, 30, 25, and 20 ms leading to a shorter total scan time of ~ 8 s.

IDEAL images were reconstructed onto a 128 *×* 128 matrix using a similar algorithm to those described in Wiesinger et al [33] and Schulte et al [29]. Image intensity variations in the PTBP and RBC compartments were analyzed selecting three ROIs, the left and right lungs with a matrix size of 80 × 40 and the heart with a matrix size of 30 × 30 (as shown in Supplementary Fig. 1). ROI-left with matrix size 80 × 40 included signal from the left lung and heart. Similarly, ROI-heart with matrix size 30 × 30 included signal from the left lung and heart. The dissolved phase ^129^Xe heart signal in ROI-left and the dissolved phase ^129^Xe lung signal in ROI-heart were separated by solving two variable equations as explained in appendix.

The normalized dissolved phase signals from ROI-left, ROI-right, and ROI-heart in the time-series PTBP and RBC images were fitted to the simple exponential function as described by Mansson et al [13, 21] as explained in Supplementary Fig. 1. Collectively, the normalized data points and the exponential fit were described as XGTD curves. The gas-transfer time constant, *τ*_*1*_, which corresponded to the rapid exponential increase, *S*_*1*_, the linear increase, and *S*_*o*_, the *y*- intersect of XGTD curve were measured in all subjects as reported in Table 1. Additionally, the gas compartment time-series images were analyzed by fitting the signal loss equation to the gas signal at right and left ROIs (as explained in Supplementary Fig. 1) as demonstrated in the appendix.

**Table 1:** Summary of patient with lung emphysema FEV_i_ -> FEV_1_

Subject number	Sex (male/female)	Age (years)	CT emphysema (% left/right)	FEV_1_(% predicted)	FEV_1_/FVC (% predicted)
1	Male	89	6/20	78	73
2	Female	74	6/7	76	85
3	Male	69	8/5	43	48
4	Female	74	2/3	92	81

*FEV*_*1*_ forced expiratory volume in 1 s, *FVC* forced vital capacity

The Welch’s *t* test in GraphPad Prism (GraphPad Prism version 7.00, GraphPad Software) was used to investigate any potential difference in the fitting parameters (i.e,. *S*_*1*_, *S*_*o*_, and *τ*_*1*_) between ROIs within the subjects with a 95% confidence interval. For proof of concept in clinical practice, fours patients currently involved in another HPX study with CT proven pulmonary emphysema and abnormal lung function were then scanned using the same technique. The CT diagnosis of emphysema was made using CT density maps, with a threshold of – 940 Hounsfield units. After optimization of gas-transfer time points from the images were obtained from a healthy subject as explained previously, XGTD curves were compared between the normal (*n* = 5) and pulmonary emphysema (*n* = 4) subjects as shown in Table 1.

## Results

**Fig. 2:**
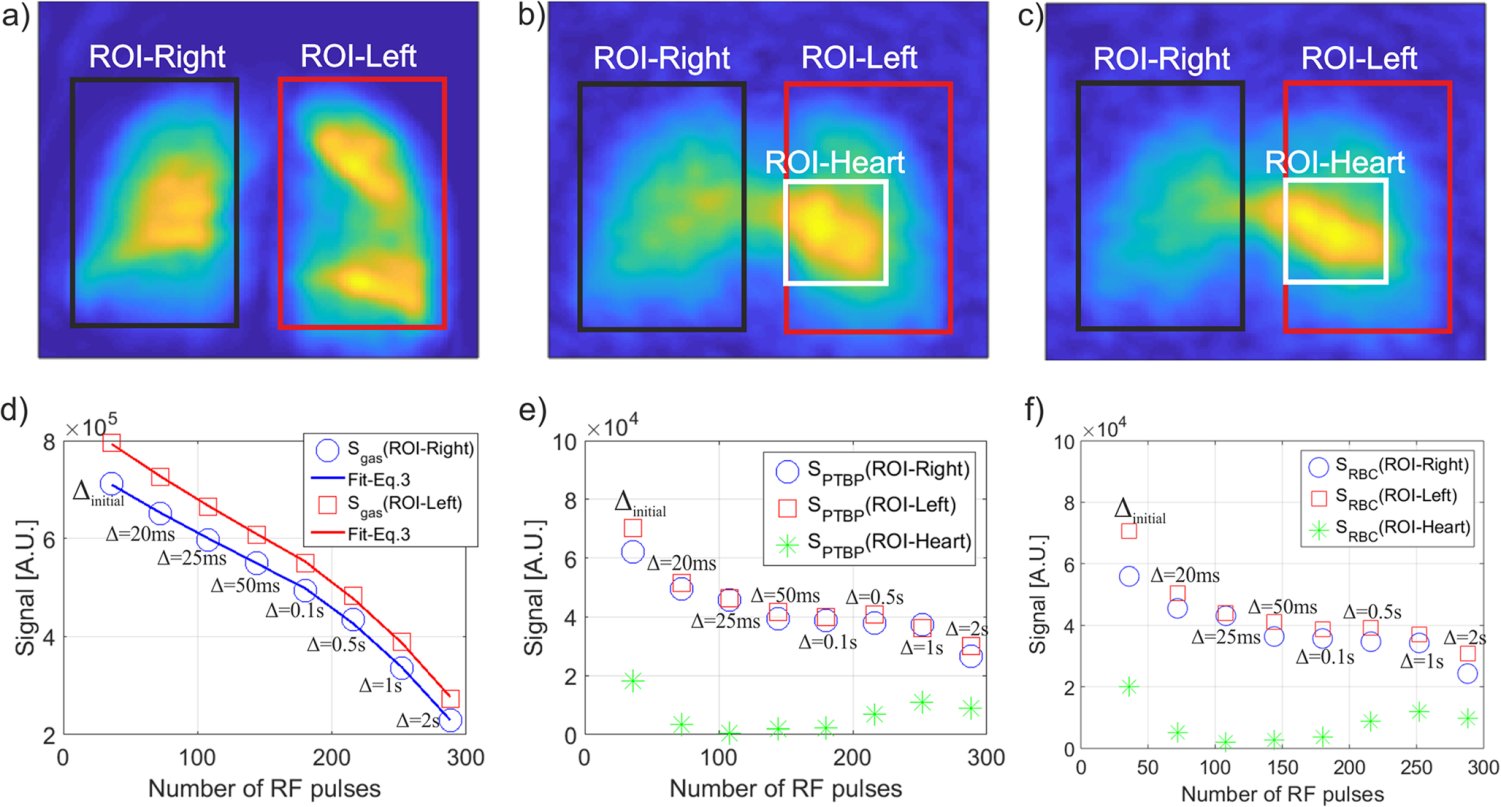
Average of time-series IDEAL images of gas, PTBP, and RBC compartments is shown in (**a**–**c**) for *∆*_late_. The signal dynamics from ROI-right, ROI-left, and ROI-heart are also shown from the gas, PTBP, and RBC compartments of the time-series images in (**d**–**f**), respectively

The averages of all time points over the *∆*_late_ in time-series IDEAL gas compartment images including ROI-Left and ROI-Right are shown in Fig. 2a. The corresponding gas signal from each time point in the time-series IDEAL gas compartment images and the corresponding fit from Eq. (3) for the ROI-left and ROI-right are shown in Fig. 2d. As expected, the S_gas_ decreases continuously during the acquisition of time-series IDEAL images as a function of *T*_1_ = 35 ± 10 s and number of RF pulses (36 per IDEAL image) with α_Gas_ = 3.6 ± 0.4°. During the five time points between *∆*_initial_ and ∆ = 0.1 s which correspond to the first 8 s of the entire scan time (17 s), the reservoir gas signal drops monotonically. However, for the later three time points between ∆ = 0.5 s and ∆ = 2 s, corresponding to the scan time interval between 8 s and 17 s, the reservoir gas signal drops more rapidly due to *T*_1_. As the scan time increases, the effect of *T*_1_ becomes more noticeable in the reservoir gas signal as also shown in Supplementary Fig. 2.

The average of PTBP and RBC compartment images of all time points over the *∆*_late_ in time-series IDEAL is shown in Fig. 2b, c. The time-series PTBP and RBC compartment imaging of the lungs are also shown for each time points in Supplementary Fig. 2. A strong signal is visible from ROI-heart (Fig. 2b, c) resulting in a different image texture compared to the gas compartment image (Fig. 2a). The temporal PTBP and RBC signal from each time point over *∆*_late_ from ROI-left, ROI-right, and ROI-heart is shown in Fig. 2e, f, respectively. The S_PTBP_(ROI-left) and S_PTBP_(ROI-right) in Fig. 2e and S_RBC_(ROI-left) and S_RBC_(ROI-right) in Fig. 2f follow a different trend than S_gas_ in Fig. 2d corresponding to the replenishment of dissolved phase signal as a function of ∆. In particular, the S_PTBP_(ROI-heart) and S_RBC_(ROI-heart) increase significantly at time points of ∆ = 0.5 s:2 s but remain low at the early time point of ∆ = 20 ms:100 ms in Fig. 2e. f. For example, S_PTBP_(ROI-heart) = 0.2 × 10^4^ is only 5% of the S_PTBP_(ROI-right) = 4.4 × 10^4^ at ∆ = 25 ms in Fig. 2e. However, Fig. 2e demonstrates S_PTBP_(ROI-heart) = 1 × 10^4^ is 42% of the S_PTBP_(ROI-right) = 2.4 × 10^4^ at ∆ = 2 s showing that the arrival of xenon from the alveoli to the heart is longer than the arrival of xenon to the pulmonary tissue and corresponding pulmonary capillary network. This difference in the arrival time of xenon in ROI-heart and ROI-right Fig. 2e, f also confirms that the separation of heart signal was effective (as explained in Appendix: *Separation of Heart Signal*). After correction of the heart signal, ROI-left and ROI-right were identical in the healthy subject as expected. The delayed arrival of xenon to the heart (ROI-heart) is also shown in Supplementary Fig. 2 in axial and coronal views confirming the delayed arrival of dissolved phase HPX to the heart for the late gas-transfer time points (> 200 ms).

**Fig. 3:**
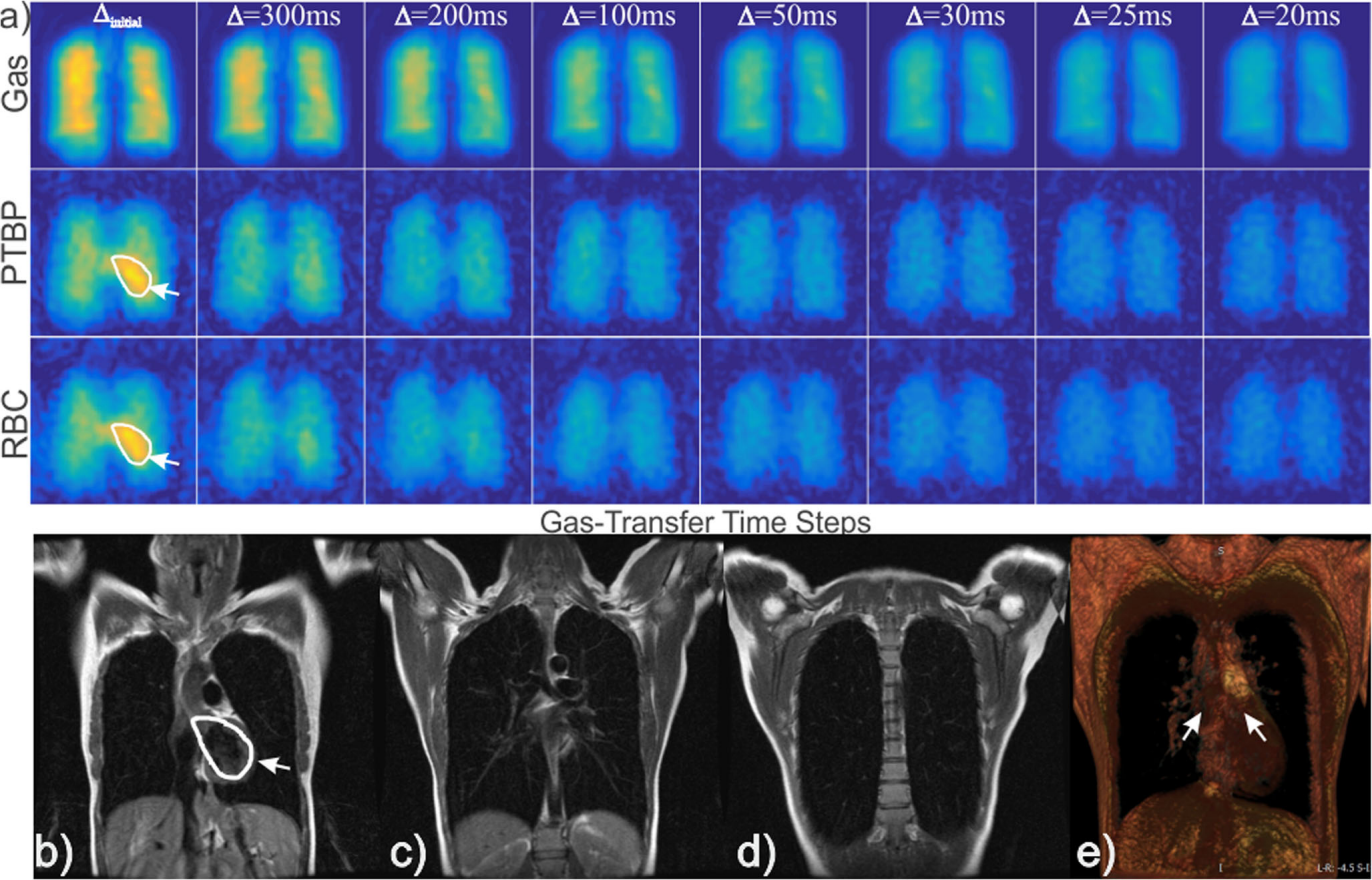
Time-series IDEAL images of gas, PTBP, and RBC compartments from a healthy subject is shown in (**a**) as a function of gas-transfer time, ∆, respectively. MRI DIR black-blood coronal images of the same subject from three slices are shown from (**b**) to (**d**) including the 3D render of six coronal slices in (**e**). ∆_initial_ is the spoiling acquisition. ROIs with arrows shows the left ventricle of the heart in (a–b). The arrows in (e) shows the pulmonary veins

The time-series IDEAL images from another healthy subject are shown in Fig. 3a in which the effect of T_1_ signal loss was minimized by capturing more short gas-transfer time points (*∆*_short_: *∆*_initial_, 300, 200, 100, 50, 30, 25, and 20 ms) resulting in a shorter scan time of ~ 8 s. The signal is strongest in the center of the PTBP and RBC compartment images at the initial acquisition, ∆_initial_, in Fig. 3a as shown with arrows. Three coronal slices of ^1^H MRI of the thorax from the same subjects are also shown in Fig. 3b–d, including the 3D render of six coronal slices in Fig. 3e. The left ventricle of the heart and the corresponding arterial vasculature vessels are shown with arrows in Fig. 3b and e, respectively. In comparison to the ^1^H MR images, the strong dissolved phase signal in the center (Fig. 3a) at ∆_initial_ is likely to have originated from the dissolved phase ^129^Xe signal in the left ventricle, where the dissolved ^129^Xe accumulated during the time between inhalation of the ^129^Xe gas and the scan starting.

**Fig. 4:**
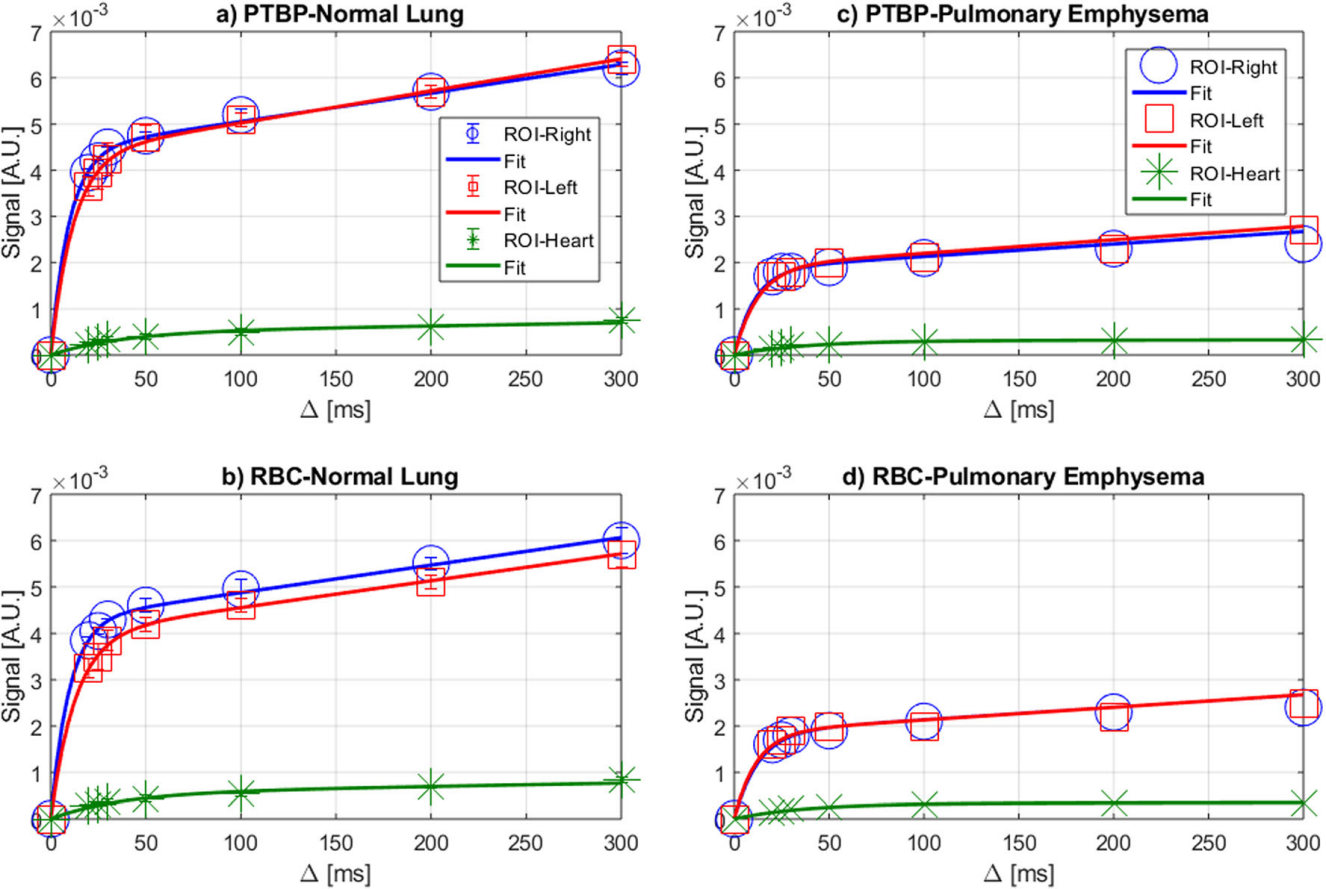
XGTD curves including the data points are shown for a normal subject and pulmonary emphysema subject (third subject in Table 1) in (**a**, **b**) and (**c**, **d**) from PTBP and RBC compartments, respectively

The mean of two XGTD curves from the same healthy subject including the standard deviation are shown in Fig. 4a, b demonstrating the separation of PTBP and RBC compartments. The average standard deviation of the repeated measurements was less than 5.2% suggesting that the repeated measurements are reasonably consistent. There was no statistical difference between the ROI-left and ROI-right (*p* value > 0.175 and *p* value > 0.128) of five healthy participants. The *τ*_*1*_ from ROI-heart (*τ*_*1*_ = 31-37 ms) demonstrated a statistical difference compared to ROI-left (*τ*_*1*_ = 9.3–13 ms) and ROI-right (*τ*_*1*_ = 9.4–13 ms) (*p* value < 0.001) and approximately three times greater than the lung signal due to the delayed arrival of dissolved phase xenon in the heart. XGTD curves from a patient with pulmonary emphysema are also shown in Fig. 4c, d. The amplitude of XGTD curves from the emphysema subjects was substantially smaller than the healthy subjects. The actual time-series IDEAL images of the healthy and the patient with emphysema are also shown in Supplementary Fig. 3.

**Fig. 5:**
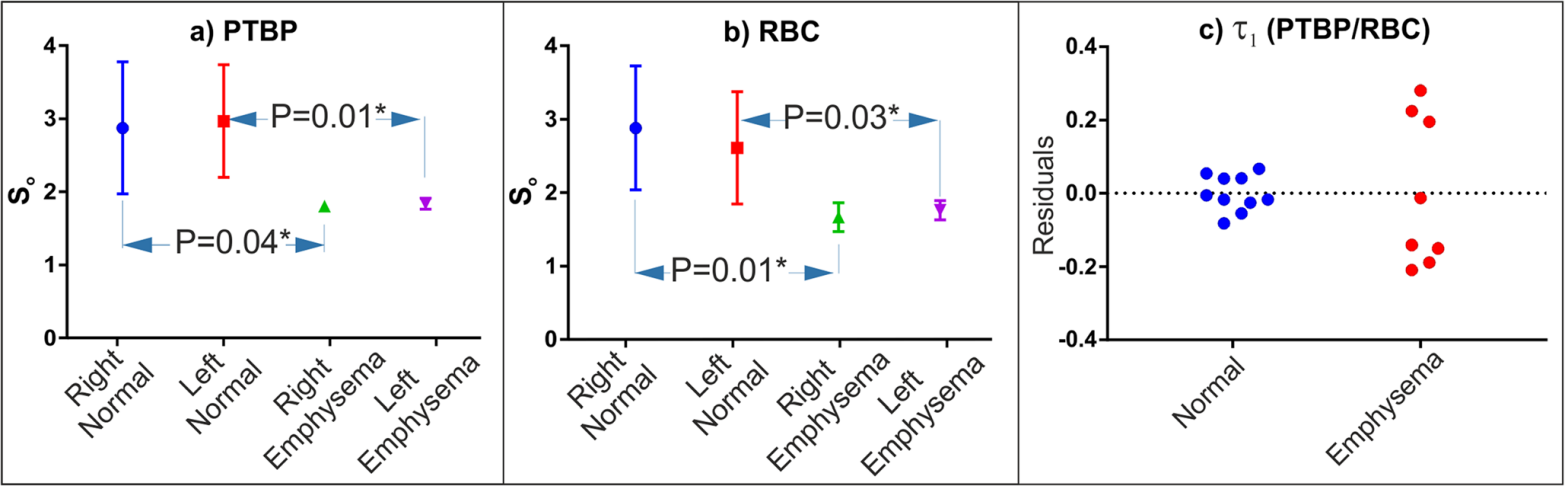
*S*_*o*_ is the *y*-intersect of the XGTD curve compared between the normal and pulmonary emphysema group separately for the PTBP and RBC compartment of the lungs in (**a**) and (**b**). P is the *p* value obtained from Welch’s *t* test and asterisks represent the statistical significance level *p* < 0.05. The residual plot of the ratio of *τ*_*1*_ between PTBP and RBC compartments is also shown in (**c**) separately for the normal and emphysema groups

The XGTD curve fitting parameters were measured with a very high fitting accuracy (*R*-squared > 0.98) and summarized in Table 2 for the normal and abnormal lungs. For a statistical comparison, *S*_*o*_ in Table 2 was compared between normal and abnormal lungs as shown in Fig. 5a–b (including the *p* values). *S*_*o*_ values of the patients with emphysema were smaller and statistically different to the normal lungs. The mean of *S*_*o*_ in the normal lungs was nearly two times greater than the emphysema group in Table 2. Interestingly, the ratio of *τ*_*1*_ between PTBP and RBC compartments was 0.97 ± 0.001 over the healthy subjects suggesting the gas transfer was repeatable in healthy subjects. However, the pulmonary emphysema patients had a greater standard deviation (1.07 ± 0.07) showing a larger variation due emphysema as shown in Fig. 5c.

**Table 2:** Summary of the XGTD curve fitting parameters in each participant

Subjects		Scans	ROI-Right			ROI-Left			ROI-Heart
			S_1_ (×10^6^)	S_o_ (×10^3^)	τ_1_ (ms)	S_1_ (×10^6^)	S_o_ (×10^3^)	τ_1_ (ms)	τ_1_ (ms)
Normal Lungs	1	PTBP	3.28	2.50	10.0	4.18	2.74	12.0	35.9
		RBC	3.11	2.34	10.5	3.18	2.33	12.6	37.8
	2	PTBP	4.34	2.84	10.7	3.56	2.55	11.3	34.6
		RBC	4.30	3.05	11.1	3.29	2.42	10.9	34.2
	3	PTBP	3.15	2.26	9.4	3.39	2.48	10.6	35.8
		RBC	3.10	2.23	10.6	3.25	2.17	10.5	35.3
	4	PTBP	6.14	4.44	9.7	6.89	4.33	11.8	37.5
		RBC	5.95	4.28	9.6	5.80	3.97	12.9	37.0
	5	PTBP	4.03	2.74	8.7	3.26	2.33	10.0	34.3
		RBC	3.36	2.51	8.5	3.12	2.17	10.6	33.7
Pulmonary Emphysema	1	PTBP	2.61	1.77	13.0	2.55	1.75	11.8	31.7
		RBC	2.41	1.58	9.6	2.12	1.57	9.3	31.5
	2	PTBP	2.76	1.85	11.5	2.63	1.80	12.0	31.2
		RBC	2.63	1.78	13.0	2.61	1.77	13.0	31.6
	3	PTBP	2.71	1.86	10.8	2.93	1.90	12.3	32.9
		RBC	2.70	1.87	12.5	2.73	1.86	11.6	33.3
	4	PTBP	2.45	1.73	12.2	2.80	1.90	8.55	34.0
		RBC	1.96	1.43	9.4	2.72	1.84	9.17	33.1

*PTBP* is pulmonary tissue and blood plasma, and *RBC* is the red blood cell compartment of lungs. S_1_, S_o_, and τ_1_ are the the fitting parameters. ROI-Right, ROI-Left, and ROI-Heart are the region of interests.

Particularly, for the subjects in the Supplementary Fig. 3, the ratio of XGTD curve fitting parameters was compared between four ROIs as shown in Sup Fig. 3 a–b, showing the feasibility of regional analysis. The ventilation and corresponding gas compartment signal were comparable between the healthy and pulmonary diseased subjects from the upper and lower right lungs (ratio of Mi = 0.88 and 1.02 in Table 3). The ratio of the XGTD curve fitting parameters (i.e., *S*_*o*_ and *S*_*1*_ in Table 3) was a factor of 5.69–2.05 higher in the healthy subject compared to pulmonary emphysema subject.

**Table 3:** Ratio of fitting parameters from four ROIs between a healthy subject and pulmonary emphysema in Supplementary Figure 3

ROIs	Gas	PTBP		RBC	
	Mi	S_1_	S_o_	S_1_	S_o_
Right upper	0.88	5.07	2.70	5.69	3.05
Right lower	1.02	3.11	4.44	3.35	2.05
Left upper	2.69	5.48	5.02	5.93	3.89
Left lower	2.04	5.09	5.36	5.84	2.55

ROIs are shown in Supplementary Fig. 3. M_i_ is a scaling factor that is dependent on the degree of polarization, concentration of HPX gas. PTBP is pulmonary tissue and blood plasma, and RBC is the red blood cell compartment of lungs. *S*_*1*_ is the linear increase and *S*_*o*_ is the y- intersect of XGTD curve

## Discussion

The multi-interleaved spiral k-space sampling IDEAL technique enabled time-series IDEAL imaging of HP ^129^Xe in the gas, PTBP, and RBC compartments in human lungs using a standard clinical MR scanner. As a proof of concept study, the feasibility of measuring the XGTD curves has been successfully demonstrated in both participant groups with normal and abnormal lungs. The proposed technique in this study showed a statistical difference between the subjects with abnormal and normal lungs. To our knowledge, this is the first in-man study showing the transfer of ^129^Xe gas in the alveoli into PTBP and RBC compartments of the lungs, and into the pulmonary vasculature and the left ventricle of the heart. Additionally, the amount of gas transfer from lung alveolar sacs to the PTBP and RBC compartments and thereby XGTD curves were impaired significantly in emphysema subjects compared to the healthy cohort.

The early XTC imaging technique is conceptually most relevant to the time-series IDEAL imaging in this study. As shown in the time-series IDEAL images the dissolved phase (PTBP and RBC), image texture is different to the gas ventilation distribution of HPX gas. Direct imaging of the dissolved phase compartments allows the capture of the diffusion of dissolved phase ^129^Xe from the lungs to the heart. Therefore, the dissolved phase HPX can be directly and quantitatively measured from the actual PTBP and RBC compartment images by selecting ROIs in the heart and lungs separately. In this respect, the time-series IDEAL imaging technique we have developed may provide insights into the measurement of XGTD similar to those of whole lung MR spectroscopy, with the added advantage of regional measurement of gas-transfer dynamics.

The measured *τ*_*1*_ reported in this study was comparable to those of measured with XTC imaging technique (~ 11 ms) [34–36] and MR spectroscopy (26 ± 17 ms) [15, 16]. The *τ*_*1*_ corresponded to ~ 50% of the dissolved phase signal compared to the dissolved phase xenon signal at ∆ = 300 ms in Fig. 4a suggesting that most of the diffusion from alveoli to the pulmonary tissue and capillaries occurs in the order of milliseconds. Additionally, the time-series IDEAL technique in this study allowed measurement of *τ*_*1*_ from the heart. Since the dissolved phase xenon at ROI-heart followed a longer pathway, the *τ*_*1*_ has been found to be approximately three times longer than that of xenon arriving to the ROI-left and ROI-right. Furthermore, the ratio of *τ*_*1*_ between PTBP and RBC compartments showed a lower variance in the heathy group than in patients with pulmonary emphysema suggesting that the emphysema affects not only the gas ventilation in the lung air sacs but also the transfer of xenon between the PTBP and RBC compartments.

The *S*_*o*_ corresponding to the *y*-intersect of the XGTC curves and dissolved phase signal amplitude was lower in the patients with lung emphysema than in the subjects with normal lungs. This is in agreement with the findings reported by Qing et al in a cohort of COPD patients [22]. Accordingly, the decrease in *S*_*o*_ may be attributed to changes caused by emphysema in the abnormal lung group. Physiologically, the decreased *S*_*o*_ would be attributed to the decreased gas transfer surface area between alveolar airs sacs to pulmonary tissue and capillary network of the lungs as a result of emphysema. The decreased gas transfer surface area is likely to be due to the loss of alveolar walls and the destruction of capillaries which may not significantly affect the gas compartment signal but can result in significant changes in XGTD curves. Therefore, in addition to the gas ventilation, the pattern of change in XGTD curves (i.e., *S*_*o*_, *S*_*1*_, and *τ*_*1*_) would allow for additional functional information to be derived and would be dependent on the type of lung disease present. This was demonstrated in the increase of dissolved phase signal associated with the increased interstitial thickening in patients with idiopathic pulmonary fibrosis [18, 37]. Furthermore, in the radiation-induced lung injury model, the XGTD curves from the PTBP and RBC compartments and apparent diffusion coefficient from HPX gas were shown to change independently due to the radiation pneumonitis and inflammation [11, 31, 38–41]. In the future, this technique (i.e., measured *S*_*o*_, *S*_*1*_, and *τ*_*1*_) may potentially be used to investigate the severity of pulmonary disease, including the quantitative measurement of lung physiological parameters (i.e., tissue thickness, capillary diffusion length, perfusion, and mean transit time) when incorporated into a suitable gas exchange numerical model.

One major limitation of our study is that the XGTD curves were not fitted to the numerical gas exchange models, although the technique was developed for quantitative measurement of lung physiology by fitting to the numerical models. The existing gas exchange models [13–15, 17, 42] cannot be directly applied to the time-series IDEAL images with multi-interleave spiral k-space acquisition for calculating the lung gas exchange parameters without a theoretical justification. As the existing models were suitable for fitting the MR spectroscopy of the whole lungs where the spatial variation in the dissolved phase images was not considered, single-shot spiral IDEAL data as the replenishment of dissolved phase magnetization due to the TR_min_ is negligible. Incorporating the fitting parameters (*S*_*o*_, *S*_*1*_, and *τ*_*1*_) reported in Table 2 in a suitable numerical gas exchange model, the lung gas exchange parameters (i.e., tissue thickness, capillary diffusion length, perfusion, and mean transit time) and variation between the same subject scans and the mean of healthy subject scan can be investigated quantitatively. Another limitation was the relatively low polarization (10–13%) limiting the voxel by voxel analysis of the XGTD particularly for the diseased subjects with short gas-transfer times (*∆ =* 20–50 ms) when the replenishment of dissolved phase magnetization is low. The signal detected should be significantly improved using higher polarization levels and better spatial and frequency selective RF pulses in the future [43].

In conclusion, the multi-interleave spiral k-space sampling IDEAL approach enabled imaging HPX in gas, PTBP, and RBC compartments in human lungs using a standard clinical strength gradient MR scanner within an easily tolerated breath-hold of 8 s. Furthermore, the feasibility of measuring the gas-transfer curves has been successfully demonstrated in five subjects with normal lungs and four subjects with pulmonary emphysema. The proposed XGTD technique suggests that time-series IDEAL imaging can capture the transfer of ^129^Xe gas in the alveoli into the PTBP and RBC compartments of the lungs and into the pulmonary vasculature and to the left ventricle of the heart. This initial small participant study has also shown that this technique may potentially differentiate between patients with normal and abnormal lungs.

## Electronic supplementary material

ESM 1 (DOCX 12527 kb)

## Abbreviations

∆: Gas-transfer time points
*∆*_initial_: Initial gas-transfer time point for spoiling the initial dissolved phase signal
*∆*_late_: Late gas-transfer time points
*∆*_short_: Short gas-transfer time points
^129^Xe: Xenon-129
COPD: Chronic obstructive pulmonary disease
HP: Hyperpolarized
HPX: Hyperpolarized xenon-129
IDEAL: Iterative decomposition of water and fat with echo asymmetry and least-square estimation
MR: Magnetic resonance
MRI: Magnetic resonance imaging
PTPB: Pulmonary tissue and plasma blood
RBC: Red blood cell
ROI: Region of interest
*S*_*1*_: The linear increase of XGTD curve
S_Gas_: Signal gas
SNR: Signal-to-noise ratio
*S*_*o*_: The *y*-intersect of XGTD curve
S_PTBP_: Signal-PTBP compartment
S_RBC_: Signal-RBC compartment
*T*_*1*_: The longitudinal decay time
*T*_*2*_^*^: Transverse signal decay time
TE-I, TE-II, and TE-III: The first, second, and third echo images
TE_1_, TE_2_, TE_3_: The first, second, and third echo times
TR: Pulse reputation times
TR_min_: The minimum TR
XGTD: Xenon-129 gas-transfer dynamics
XTC: Xenon polarization transfer constant
α_Gas_: Flip angle on gas phase
τ_1_: The gas-transfer time constant

## References

[1] Kirby M, Svenningsen S, Owrangi A (2012). Hyperpolarized He-3 and Xe-129 MR imaging in healthy volunteers and patients with chronic obstructive pulmonary disease. Radiology.

[2] Driehuys B, Martinez-Jimenez S, Cleveland ZI (2012). Chronic obstructive pulmonary disease: safety and tolerability of hyperpolarized 129Xe MR imaging in healthy volunteers and patients. Radiology.

[3] Shukla Y, Wheatley A, Kirby M (2012). Hyperpolarized 129Xe magnetic resonance imaging: tolerability in healthy volunteers and subjects with pulmonary disease. Acad Radiol.

[4] Svenningsen S, Guo F, Kirby M (2014). Pulmonary functional magnetic resonance imaging: asthma temporal-spatial maps. Acad Radiol.

[5] He M, Driehuys B, Que LG, Huang YT (2016). Using hyperpolarized 129Xe MRI to quantify the pulmonary ventilation distribution. Acad Radiol.

[6] Matin TN, Rahman N, Nickol AH (2017). Chronic obstructive pulmonary disease: lobar analysis with hyperpolarized 129Xe MR imaging. Radiology.

[7] Ebner L, Kammerman J, Driehuys B, Schiebler ML, Cadman RV, Fain SB (2017). The role of hyperpolarized 129xenon in MR imaging of pulmonary function. Eur J Radiol.

[8] Doganay O, Matin TN, Mcintyre A et al (2018) Fast dynamic ventilation MRI of hyperpolarized (129) Xe using spiral imaging. Magn Reson Med 79:2597–2606 10.1002/mrm.26912 PMC5836876 28921655

[9] Mugler JP, Driehuys B, Brookeman JR (1997). MR imaging and spectroscopy using hyperpolarized 129Xe gas: preliminary human results. Magn Reson Med.

[10] Stewart NJ, Leung G, Norquay G et al (2014) Experimental validation of the hyperpolarized (129) Xe chemical shift saturation recovery technique in healthy volunteers and subjects with interstitial lung disease. Magn Reson Med. 10.1002/mrm.25400 10.1002/mrm.25400 25106025

[11] Fox MS, Ouriadov A, Thind K et al (2014) Detection of radiation induced lung injury in rats using dynamic hyperpolarized (129)Xe magnetic resonance spectroscopy. Med Phys 41 10.1118/1.4881523 24989401

[12] Wang Z, Robertson SH, Wang J et al (2017) Quantitative analysis of hyperpolarized ^129^ Xe gas transfer MRI. Med Phys. 10.1002/mp.12264 10.1002/mp.12264 28382694

[13] Månsson S, Wolber J, Driehuys B, Wollmer P, Golman K (2003) Characterization of diffusing capacity and perfusion of the rat lung in a lipopolysaccaride disease model using hyperpolarized 129Xe. Magn Reson Med 50:1170–1179 10.1002/mrm.10649 14648564

[14] Patz S, Muradyan I, Hrovat MI et al (2011) Diffusion of hyperpolarized 129Xe in the lung: a simplified model of ^129^Xe septal uptake and experimental results. New J Phys 13

[15] Chang YV (2013). MOXE: a model of gas exchange for hyperpolarized 129Xe magnetic resonance of the lung. Magn Reson Med.

[16] Chang YV, Quirk JD, Ruset IC, Atkinson JJ, Hersman FW, Woods JC (2014). Quantification of human lung structure and physiology using hyperpolarized 129Xe. Magn Reson Med.

[17] Stewart NJ, Parra-Robles J, Wild JM (2016). Finite element modeling of (129)Xe diffusive gas exchange NMR in the human alveoli. J Magn Reson.

[18] Wang JM, Robertson SH, Wang Z (2018). Using hyperpolarized (129)Xe MRI to quantify regional gas transfer in idiopathic pulmonary fibrosis. Thorax.

[19] Kaushik SS, Freeman MS, Cleveland ZI (2013). Probing the regional distribution of pulmonary gas exchange through single-breath gas- and dissolved-phase 129Xe MR imaging. J Appl Physiol (1985).

[20] Cleveland ZI, Cofer GP, Metz G (2010). Hyperpolarized Xe MR imaging of alveolar gas uptake in humans. PLoS One.

[21] Driehuys B, Cofer GP, Pollaro J, Mackel JB, Hedlund LW, Johnson GA (2006). Imaging alveolar-capillary gas transfer using hyperpolarized 129Xe MRI. Proc Natl Acad Sci U S A.

[22] Qing K, Mugler JP 3rd, Altes TA et al (2014) Assessment of lung function in asthma and COPD using hyperpolarized 129Xe chemical shift saturation recovery spectroscopy and dissolved-phase MRI. NMR Biomed 27:1490–1501 10.1002/nbm.3179 PMC4233004 25146558

[23] Qing K, Ruppert K, Jiang Y (2014). Regional mapping of gas uptake by blood and tissue in the human lung using hyperpolarized xenon-129 MRI. J Magn Reson Imaging.

[24] Ruppert K, Mata JF, Brookeman JR, Hagspiel KD, Mugler JP (2004). Exploring lung function with hyperpolarized (129)Xe nuclear magnetic resonance. Magn Reson Med.

[25] Ruppert K, Brookeman JR, Hagspiel KD, Driehuys B, Mugler JP (2000). NMR of hyperpolarized (129)Xe in the canine chest: spectral dynamics during a breath-hold. NMR Biomed.

[26] Muradyan I, Butler JP, Dabaghyan M (2013). Single-breath xenon polarization transfer contrast (SB-XTC): implementation and initial results in healthy humans. J Magn Reson Imaging.

[27] Patz S, Muradian I, Hrovat MI et al (2008) Human pulmonary imaging and spectroscopy with hyperpolarized 129Xe at 0.2T. Acad Radiol 15:713–727 10.1016/j.acra.2008.01.008 PMC2475597 18486008

[28] Reeder SB, Pineda AR, Wen Z (2005). Iterative decomposition of water and fat with echo asymmetry and least-squares estimation (IDEAL): application with fast spin-echo imaging. Magn Reson Med.

[29] Schulte RF, Sperl JI, Weidl E (2013). Saturation-recovery metabolic-exchange rate imaging with hyperpolarized [1-13C] pyruvate using spectral-spatial excitation. Magn Reson Med.

[30] Doganay O, Wade T, Hegarty E, McKenzie C, Schulte RF, Santyr GE (2016). Hyperpolarized (129) Xe imaging of the rat lung using spiral IDEAL. Magn Reson Med.

[31] Doganay O, Stirrat E, McKenzie C, Schulte RF, Santyr GE (2016). Quantification of regional early stage gas exchange changes using hyperpolarized (129)Xe MRI in a rat model of radiation-induced lung injury. Med Phys.

[32] Zanette B, Stirrat E, Jelveh S, Hope A, Santyr G (2017) Physiological gas exchange mapping of hyperpolarized (129) Xe using spiral-IDEAL and MOXE in a model of regional radiation-induced lung injury. Med Phys. 10.1002/mp.12730 10.1002/mp.12730 29238999

[33] Wiesinger F, Weidl E, Menzel MI (2012). IDEAL spiral CSI for dynamic metabolic MR imaging of hyperpolarized [1-13C]pyruvate. Magn Reson Med.

[34] Dregely I, Ruset IC, Mata JF (2012). Multiple-exchange-time xenon polarization transfer contrast (MXTC) MRI: initial results in animals and healthy volunteers. Magn Reson Med.

[35] Dregely I, Mugler JP, Ruset IC (2011). Hyperpolarized Xenon-129 gas-exchange imaging of lung microstructure: first case studies in subjects with obstructive lung disease. J Magn Reson Imaging.

[36] Ruppert K, Brookeman JR, Hagspiel KD, Mugler JP (2000). Probing lung physiology with xenon polarization transfer contrast (XTC). Magn Reson Med.

[37] Kaushik SS, Freeman MS, Yoon SW (2014). Measuring diffusion limitation with a perfusion-limited gas--hyperpolarized 129Xe gas-transfer spectroscopy in patients with idiopathic pulmonary fibrosis. J Appl Physiol (1985).

[38] Santyr G, Fox M, Thind K (2014). Anatomical, functional and metabolic imaging of radiation-induced lung injury using hyperpolarized MRI. NMR Biomed.

[39] Zanette B, Stirrat E, Jelveh S, Hope A, Santyr G (2017). Detection of regional radiation-induced lung injury using hyperpolarized 129 Xe chemical shift imaging in a rat model involving partial lung irradiation: proof-of-concept demonstration. Adv Radiat Oncol.

[40] Ouriadov A, Fox M, Hegarty E, Parraga G, Wong E, Santyr EG (2016) Early stage radiation-induced lung injury detected using hyperpolarized (129) Xe morphometry: proof-of-concept demonstration in a rat model. Magn Reson Med Sci 75:2421–2431 10.1002/mrm.25825 26154889

[41] Li H, Zhang Z, Zhao X, Sun X, Ye C, Zhou X (2016). Quantitative evaluation of radiation-induced lung injury with hyperpolarized xenon magnetic resonance. Magn Reson Med.

[42] Doganay O, Fox M, Santyr GE (2014) Measurement of pulmonary perfusion and gas exchange using hyperpolarized ^129^Xe in a rodent model of radiation-induced lung Injury. Proceedings of the 22th annual meeting of ISMRM (abstract ID 2290), Milan, Italy

[43] Leung G, Norquay G, Schulte RF, Wild JM (2015). Radiofrequency pulse design for the selective excitation of dissolved 129Xe. Magn Reson Med.

