# Supplementary material for: Magnetic resonance imaging of the time course of hyperpolarized 129Xe gas exchange in the human lungs and heart
Source: Eur Radiol. 2018 Dec 5;29(5):2283–92. doi: 10.1007/s00330-018-5853-9 (PMC6443604; doi:10.1007/s00330-018-5853-9)
Supplement: Supplementary file 1 — (DOCX 12527 kb) [file 330_2018_5853_MOESM1_ESM.docx]

**Appendix:**

**Separation of Heart Signal:** The dissolved phase ^129^Xe heart-signal in ROI-Left and the dissolved phase ^129^Xe lung-signal in ROI-Heart were separated by solving the simple two variable equations:

| S(ROI-Left)=S_Lung_(80×40)+S_Heart_ | [1] |
| --- | --- |
| S(ROI-Heart)=S_Lung_(30×30)+S_Heart_ | [2] |

Where S(ROI-Left) and S(ROI-Heart) are the sum of the signal in the IDEAL image within the ROI-Left and ROI-Heart; S_Lung_(80×40) is the sum of the lung signal excluding the signal from the heart and S_Heart_ is the heart signal in the ROI-Left; S_Lung_(30×30) is the sum of lung signal in the ROI-Heart. The lung signal from the ROI-Left excluding the heart and the heart signal from the ROI-Heart excluding the lung signal were calculated from Equations [1] and [2].

**HP ^129^Xe gas signal loss equation:** The gas compartment times-series images were analyzed by fitting the signal loss equation to the gas signal from the right and left ROIs (Supplementary Figure 1a) by fitting the equation to the times-series gas images.

| S_Gas_ (n, t, T_1,_ α_Gas_) = M_i_ $e^{(-t/T_{1})}$cos(α_Gas_)^n-1^sin(α_Gas_) | [3] |
| --- | --- |

Where M_i_ is a scaling factor that is dependent on the degree of polarization, concentration of HPX gas, coil sensitivity, digital filter and amplifier; T_1_ is the longitudinal polarization relaxation time; α_Gas_ is the flip angle in the gas phase; n is the number of RF pulses. The α_Gas_, T_1_, and M_i_ were calculated by fitting Eq. 3 to the time-series IDEAL gas compartment images, reported in Table 1 and used for normalization of the gas transfer curves.

**
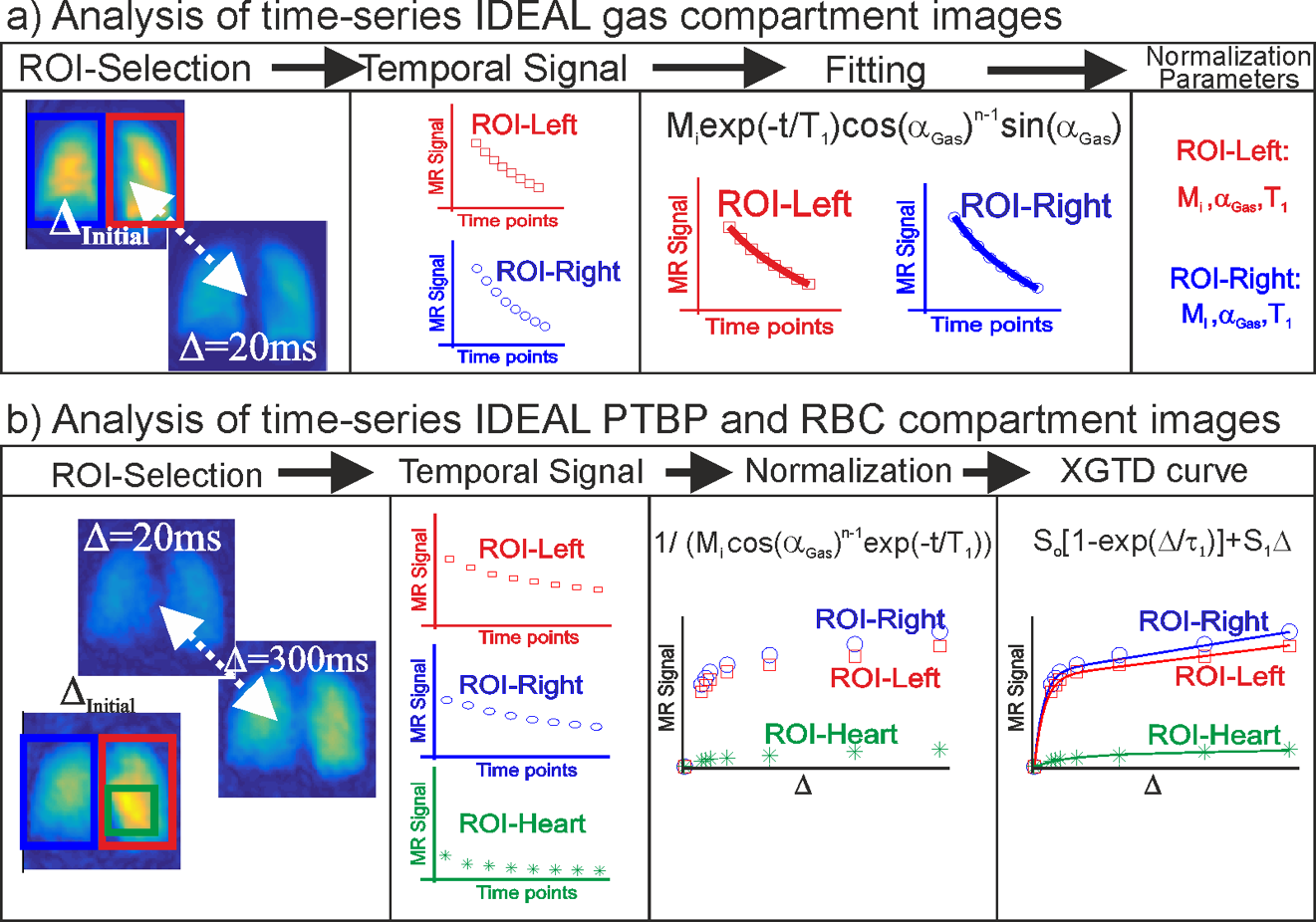
**

**Supplementary Figure 1:** Diagram of data analysis for the time-series gas compartment images and time-series dissolved phase compartment images are shown in a) and b).

**
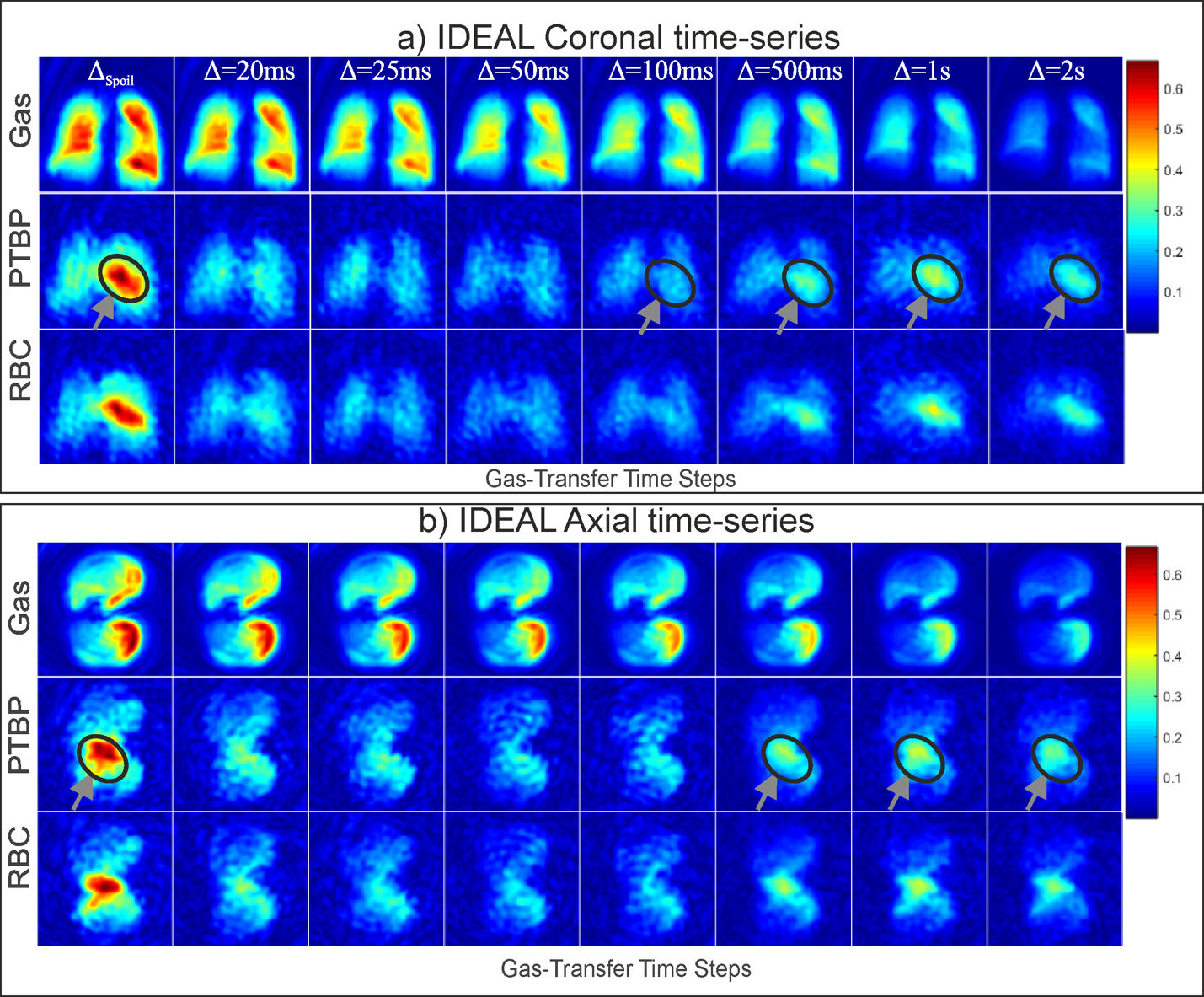
Supporting Figure 2:** Coronal and axial plane time-series IDEAL images of a normal subject with late ∆ points ***∆***_late_: ***∆***_initial_, 20, 25, 50, 100, 500, 1000, 2000 ms are shown in a) and b) respectively. Arrows show the dissolved phase xenon signal from the heart.

**
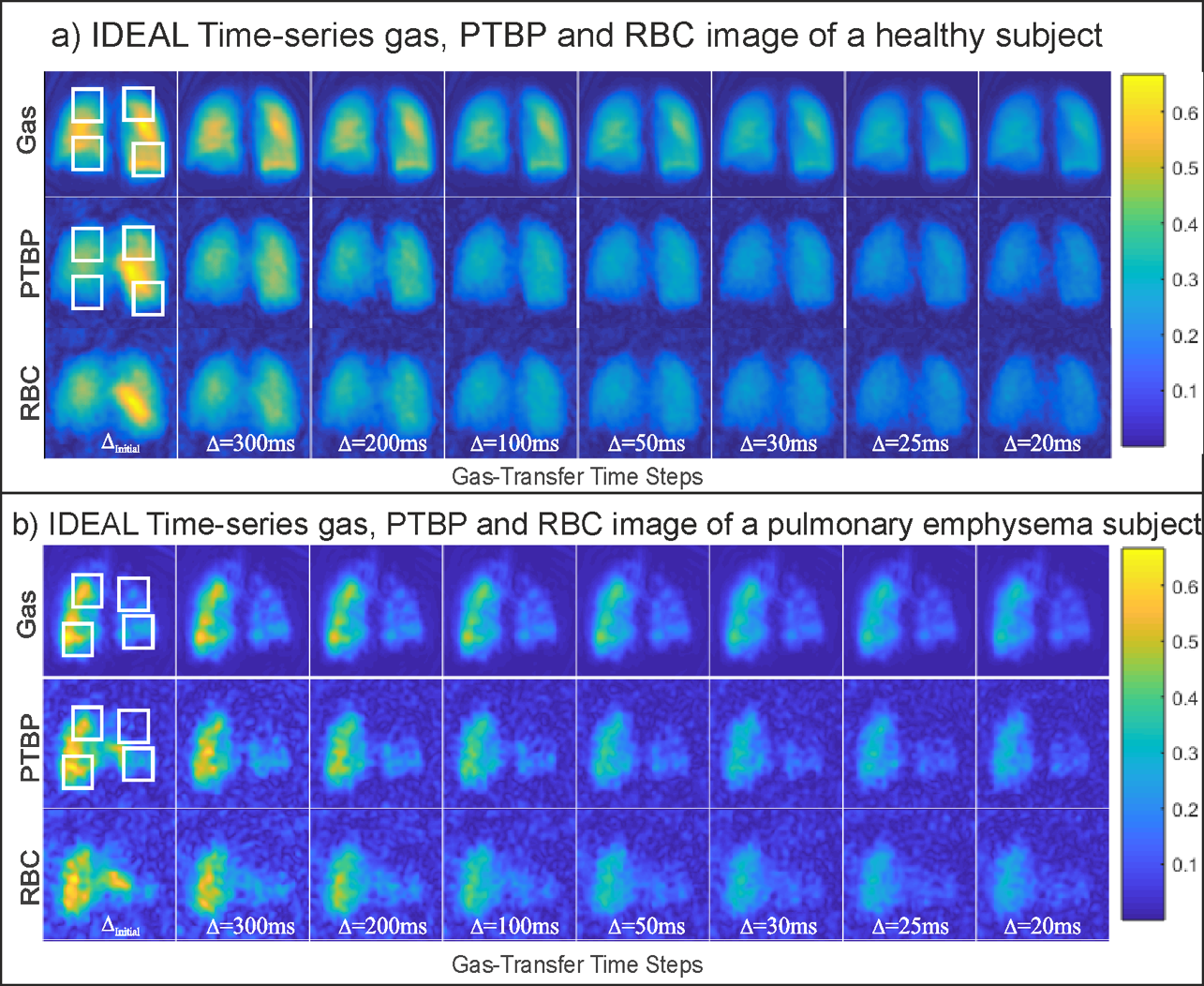
Supporting Figure 3:** Time-series IDEAL gas, PTBP and RBC compartment images are shown from a normal and pulmonary emphysema subject (3^rd^ subject in Table 1) in a) and b), respectively. Four ROIs selected for the analysis in Table 3 from the right upper and right lower, and left upper and left lower sections of the lungs are indicated.
